# Temporal Bell inequalities in cognition

**DOI:** 10.3758/s13423-023-02275-5

**Published:** 2023-04-17

**Authors:** Oliver J. Waddup, James M. Yearsley, Pawel Blasiak, Emmanuel M. Pothos

**Affiliations:** 1 Department of Psychology, City, University of London, London, UK; 2 Institute of Nuclear Physics, Polish Academy of Sciences, Kraków, Poland

**Keywords:** Constructive memory, Temporal Bell inequality, Change judgments, Quantum theory

## Abstract

There is widespread evidence that human memory is constructive, so that recollective processes may alter the information retrieved or impact on subsequent recollections. We examine a framework for narrowing down the nature of such processes, from physics. In Physics, the Temporal Bell (TB) inequality offers a general test of the sensitivity of the context of previous measurements in sequential measurement scenarios, as predicted by quantum theory. We present an empirical memory paradigm that allows a test of the TB inequality, using a novel kind of “change judgment,” whereby participants are asked to decide whether there has been a change in a question across different time points of a scenario. Across two experiments, we were able to observe evidence for the violation of a TB inequality in one case, offering evidence for quantum-like processes in memory. The present results complement other recent work purporting the relevance of quantum-like representations in memory and raise questions regarding the adaptive value of such representations.

**Supplementary Information:**

The online version contains supplementary material available at 10.3758/s13423-023-02275-5.

## Introduction

“Memory is inherently a reconstructive process, whereby we piece together the past to form a coherent narrative that becomes our autobiography” (Bernstein & Loftus, 2009, p.373). The purpose of this work is to offer a novel perspective to this claim. There is evidence that memory has a reconstructive element, so that recollection is in part faithful retrieval, in part a “filling in” of details, inferred from summary/ abstract representations. Fuzzy trace theory is the idea that memories are composed of a “verbatim” component, which offers a representation of information as faithful as possible to a target event and a “gist” component, which is comprised of summary information for the event (Reyna, 2008; Reyna & Brainerd, 1998). Such summary information can be queried flexibly, depending on specific needs, but also possibly lead to apparent false memories (Howe, 2011) and distortions (Schacter et al., 2011). Any reconstructive processes in recall are likely to depend on the particulars of the individual at the time of the recall, including the individual’s state of mind, knowledge, perspective etc., in a way analogous to how memory encoding has been suggested to depend on knowledge at the time of encoding (Nelson & Shiffrin, 2013).

Much effort has been directed towards whether false memories might have adaptive value, for example, if they create a more positive impression of one’s self or improve future tasks (Howe, 2011; Schacter et al., 2011). Another adaptive perspective is that it is hard to see how human memory could work *differently*, because, at the time of encoding, it seems impossible to anticipate all future uses of some information. We would either need to encode all possible aspects of events or utilize an encoding scheme that allows flexible subsequent querying. It seems straightforward to suggest that, whatever else the purpose of a reconstructive memory, part of it is informational efficiency. Based on these insights and previous work, is it possible to develop more precise proposals for memory representations, consistent with a reconstructive component?

Manning (2021) recently offered such a proposal, in claiming that retrieval is “conceptually more like we are simultaneously visiting many of our prior experiences, analogous to a quantum wave function spreading its probability mass over space” (p.712). The idea that recollection is not a process that can (easily) be focused on specific timepoints resonates with the notion of quantum-like waves, the characteristic of which is that probability is spread out, with “measurements” being the main way to attain sharper focus. More generally, quantum-like representations offer a promising avenue for understanding reconstructive processes, because they can be queried flexibly.

**Fig. 1:**
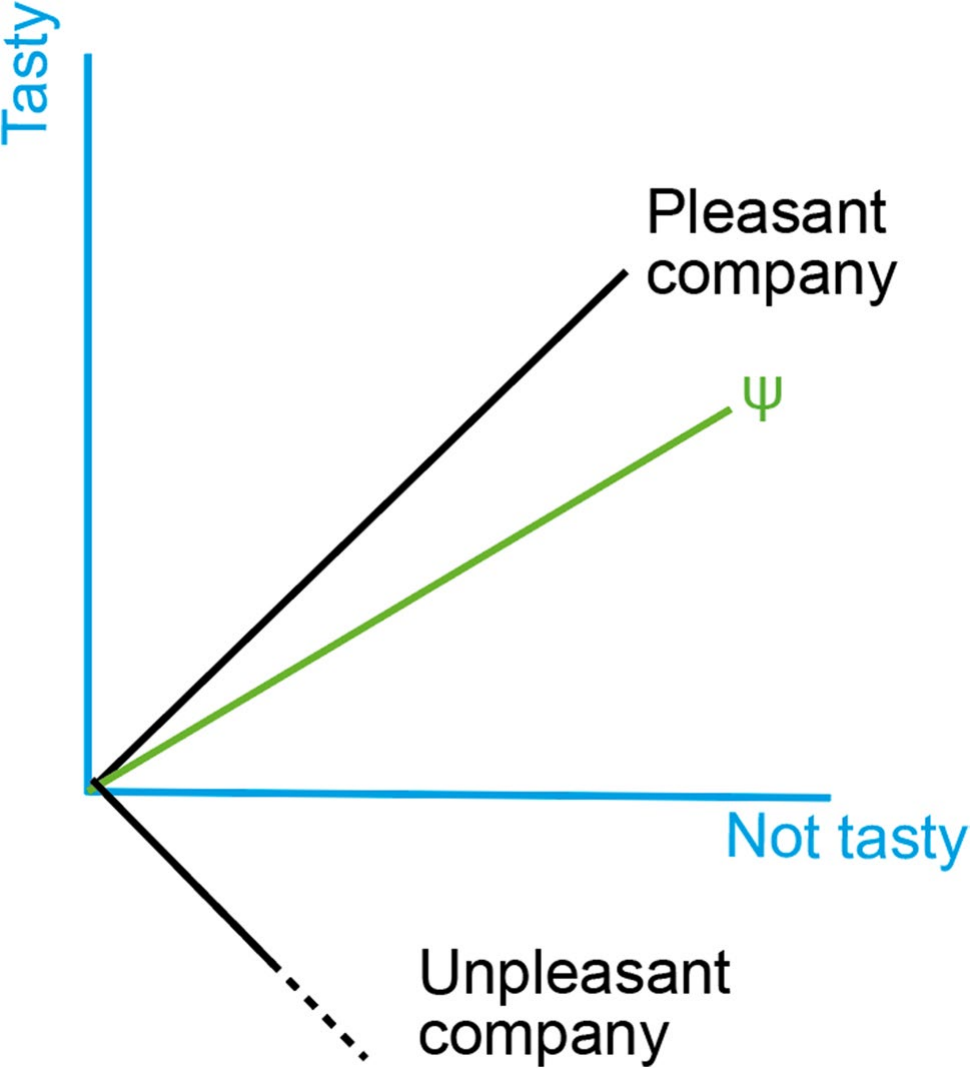
Memory as quantum-like snapshots. A quantum-like memory representation, *ψ*, can be multiply queried

Quantum-like representations in memory could look as in Fig. 1, whereby the vector labelled *ψ* represents a memory of having breakfast. In this caricature, two-dimensional example, different questions are represented as different sets of basis vectors. We show two such questions, first, whether the breakfast was tasty and, second, whether the company was pleasant. Resolving a question is a process of projection, so that higher overlap with a particular basis vector indicates higher probability of a corresponding answer. The vector *ψ* was placed close to the basis vector for “yes” to the question about the company being pleasant, to reflect the corresponding fact. Given this memory representation, we can ask either of the two questions (or indeed any possible question). Every time a question is resolved, the memory representation changes to identify with the outcome of the question. For example, if we try to recollect whether the company was pleasant or not and decide for the former, the memory representation will change to coincide with the basis vector for the pleasant company answer.

Our purpose is to offer a general test of quantum-like structure in memory. To accomplish this, we need to introduce the notion of the Leggett-Garg or Temporal Bell (TB; Leggett & Garg, 1985) inequality.

## The Temporal Bell inequality

Physicists have asked whether there is a general test of quantum structure in nature, which eschews the details of any specific model or experimental set up. Note, in behavioral sciences, at most we can talk about quantum-like structure, since the evidence indicates that brain neurophysiology is classical (Litt et al., 2006). The essential ideas concerning TB tests translate well from physics to psychology (Asano et al., 2014; Atmanspacher & Filk, 2010; Yearsley & Pothos, 2014). An “observable” is any quantity which can be observed, for example, position or momentum, or, in behavioral sciences, a question, like whether breakfast on Sunday morning was tasty. A measurement is an operation that queries a system of interest, regarding an observable. In behavioral sciences, we could ask ourselves, was our breakfast on Sunday tasty?

Macrorealism is the assumption in physics that an observable has a value, *independent of measurement*, at any given point (we retain the label macrorealism, bearing in mind that the distinction between macro and micro is not presently relevant). We can still have uncertain states, for example, linear mixtures such as 35% yes and 65% no, but the proportions could be accessible to measurement without necessarily changing the system. The quantum position is that we can have quantum-like states, *superpositions*, such that without a measurement we cannot say that the observable has any specific value. A key aspect of quantum structure is that measurements can change the state.

There are different ways in which a measurement could change a state. Noninvasive measurability (NIM) means that the measurement does not change the system.^1^ So, if we think there might be quantum structure, this assumption is problematic. The trick is to construct measurement approaches, such that we can separate out constructive influences due to quantum processes from ones which disturb the system because they might be too crude.

Finally, the arrow of time assumption is that whatever happens earlier can influence what might happen later. This is a trivial assumption, which we do not consider further.

Consider a question concerning the guilt or innocence of a suspect in a hypothetical crime. A participant in a psychology experiment receives information across consecutive time points, concerning the guilt or innocence of the suspect. The expectation of the question outcome (is the suspect guilty or not?), at two time points *i*, *j*, is given as:

1 $$\left\langle {Q}_i{Q}_j\right\rangle =\sum\nolimits_{n_i,{n}_j}q\left({n}_i\right)q\left({n}_j\right)P\left({n}_i,{n}_j\right)$$

where *n*_*i*_, *n*_*j*_ = {*guilty*, *innocent*} and we arbitrarily set *q*(*guilty*) = 1, *q*(*innocent*) =  − 1. It is straightforward to see that for bivalued questions the expectation value is like a correlation, so we can employ the notation *C*_*ij*_ = 〈*Q*_*i*_*Q*_*j*_〉. Leggett and Garg’s (1985) seminal result is that the assumptions of macrorealism, NIM, and arrow of time allow us to constrain the way question values correlate across different time points. For three time points, the most common form the Leggett-Garg or TB inequality is (note, *a*<*b*<*c*):

2 $${C}_{ab}+{C}_{bc}\le {C}_{ac}+1$$

Thus, according to the TB inequality, correlations between *successive* time points cannot exceed a bound set by the correlation between the *extreme* time points. An alternative form of the TB inequality is as follows. Suppose that macrorealism holds. Then, we can construct a table with all possible combinations of guilt values, for the suspect, across the three time points and observe when there is change in value. Indicating the number of changes in the judgment between time points *i*, *j* as *N*_−_(*t*_*i*_, *t*_*j*_), with straightforward set theory, we can show that *N*_−_(*t*_1_, *t*_3_) ≤ *N*_−_(*t*_1_, *t*_2_) + *N*_−_(*t*_2_, *t*_3_). This inequality is equivalent to the TB one (Atmanspacher & Filk, 2010; Online Supplementary Material (OSM) 1) and, surprisingly, without macrorealism, it does not hold. The singular accomplishment of the TB inequality is that it offers a route to test macrorealism.

**Fig. 2:**
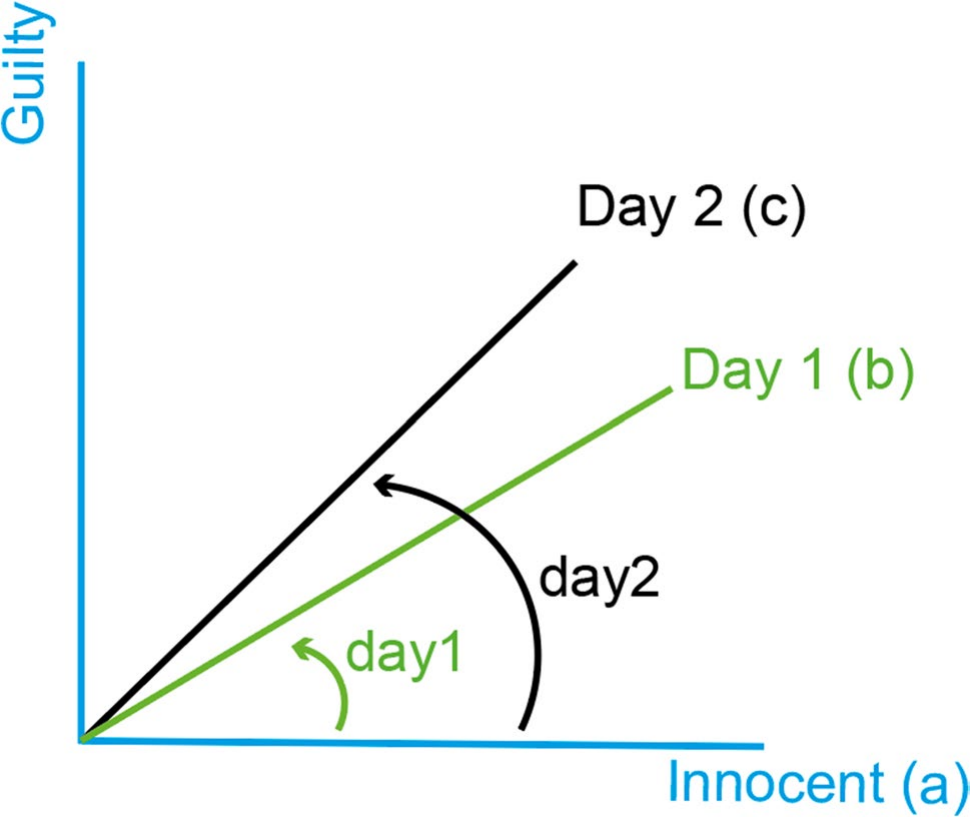
A caricature quantum model for violating the Temporal Bell inequality

A caricature quantum model which violates the TB inequality is shown in Fig. 2. Assume that the suspect is initially considered innocent (imagine the memory state along the Innocent ray) and that on two successive days of trial proceedings incriminating evidence is presented. Then, both *C*_*start*, *day*1_ and *C*_*day*1, *day*2_ would be relatively high, but *C*_*start*, *day*2_ would be low enough to violate the TB inequality (OSM 2).

Testing macrorealism through the TB inequality has an almost magical quality. There are four points to consider regarding psychological relevance. First, why does it matter whether uncertainty is in the form of classical linear mixtures (such as 35% guilty and 65% innocent) versus superpositions? This is because in the former case there are no firm requirements regarding introspective access to the relevant proportions or how a measurement (question) could change the underlying mental state. With superpositions, prior to a measurement, we cannot say that the individual has any belief regarding guilt or innocence and a decision has to “construct” a state that is either pure guilt or pure innocence. With superpositions, it is impossible to construct a joint probability distribution for question values at different time points: there is no classical trajectory (Atmanspacher & Filk, 2010; Yearsley & Pothos, 2014). The TB inequality offers a general test for the existence of superpositions versus linear mixtures.

Second, the claim that with superpositions measurements (e.g., recollections) can change the mental state might appear not unique to quantum theory: behaviorally, there is evidence that judgments construct, rather than reveal, preferences, opinions, etc. (Schwarz, 2007; Sharot et al., 2010). In memory, it appears unlikely that memories exist independently of whether we query them, rather questions help construct what we experience in recollection (cf. Manning, 2021). The NIM assumption in a TB inequality test can help separate constructive influences specific to quantum-like processes from ones from other sources, for example, ‘crude’ measurements. So, a TB inequality test can narrow down the nature of constructive influences in memory.

Third, in recent years there have been several proposals of quantum cognitive models, including in memory, for example, as instantiations of verbatim versus gist representations (Brainerd et al., 2013; Denolf & Lambert-Mogiliansky, 2016; Trueblood & Hemmer, 2017). Such models assume that mental states are superpositions (Busemeyer & Bruza, 2011; Pothos & Busemeyer, 2022). The TB inequality offers a general corresponding test. Evidence for superpositions goes hand in hand with several implications, such as, (as noted) constructive influences, interference effects, order effects, contextuality etc. Concerning contextuality, consider a question (such as whether a suspect is guilty) at different time points. If a person recalls whether the suspect was guilty at *t*_1_, this creates a unique context for answering at other times versus if there is no recollection at *t*_1_. That is, resolving a question interferes with how it is resolved at other times. This effect is identical to how quantum-like models explain fallacies, like the conjunction fallacy, whereby participants consider Linda more likely to be a feminist and bank teller than just a bank teller. The quantum-like explanation is that answering the feminist question first (in the conjunction) creates a unique perspective for resolving the bank teller one (Busemeyer et al., 2011; Pothos et al., 2017).

Finally, superpositions may resolve the challenge of information overload in memory encoding. Quantum-like representations allow encoding of events as *snapshots*, which we can employ later for any question. The price for such efficiency is that every time a question is resolved, the memory representation has to change to identify with the outcome of the question (cf. Manning, 2021), which impacts on subsequent recollections.

## Methods

### Participants

The two experiments are described together. Exploratory sample sizes were 400 for both experiments, recruiting in Experiment 1 and Experiment 2, respectively, 401 participants (196 males, 201 females, two non-binary, and two not responded) and 409 participants (202 males, 205 females, two non-binary), via Prolific Academic and mTurk (requesting “Master workers”). Experiment 2 sampling was carried out in two stages (January 2022, August 2022). Most participants reported very high fluency in English.

### Materials and procedure

Participants were told they were jurors in a trial of Mr. Smith, a suspect in a hypothetical murder mystery. They were told to assume that Smith is initially innocent and to consider prosecution evidence across the two days in the trial. The victim, Mr. Dixon, had been sharing an apartment with Mr. Smith, until the former’s death (scripts are available in the Open Science Framework (OSF) page for the project, https://osf.io/g57vb/). Dixon was found in his bed, apparently having died from an overdose of sleeping pills. The prosecution claims that Smith slipped the pills into the glass Dixon was drinking from, while the defense claims that Dixon deliberately took an overdose. Table 1 shows the evidence presented on each of the 2 days.

**Table 1:** Prosecution evidence in Mr. Smith’s hypothetical trial

Evidence	Day 1	Day 2
Experiment 1	The addition of the sleeping pills to the liquor was unlikely to have altered its taste.	Smith's fingerprints were found on the bottle of liquor at Dixon's bedside.
Experiment 2	Smith had a previous conviction for violent disorder.	Smith's fingerprints were found on the bottle of liquor at Dixon's bedside.

To assess putative TB inequality violations, each participant needs to contribute data to evaluate any single one of the quantities *C*_*ab*_, *C*_*bc*_, or *C*_*ac*_ in Eq. 2, which, recall, are expectation values, e.g., *C*_*ab*_ = *p*(++| *ab*) + *p*(−−| *ab*) − *p*(+−| *ab*) − *p*(−+| *ab*). So, to compute *C*_*ab*_, we need to measure the conjunction of different outcomes of the question (‘is Smith guilty?’) at time point *a* and time point *b*. The difficulty in physical tests of the TB inequality is that an early measurement may disturb the system either because of quantum processes or “coarse” measurements, hence forcing us to reject the NIM assumption (Emary, 2017; Wilde & Mizel, 2012). Ideally, we want to measure at time point *b*, without having measured at *a* first, but of course, in physics, this is impossible, if we are to compute conjunctions. Interestingly, in behavioral settings, this fundamental difficulty can be circumvented, by employing *change* measurements, that is, asking for whether the value of a question has changed, *without* asking for the question value at the two time points (OSM 1).

**Fig. 3:**
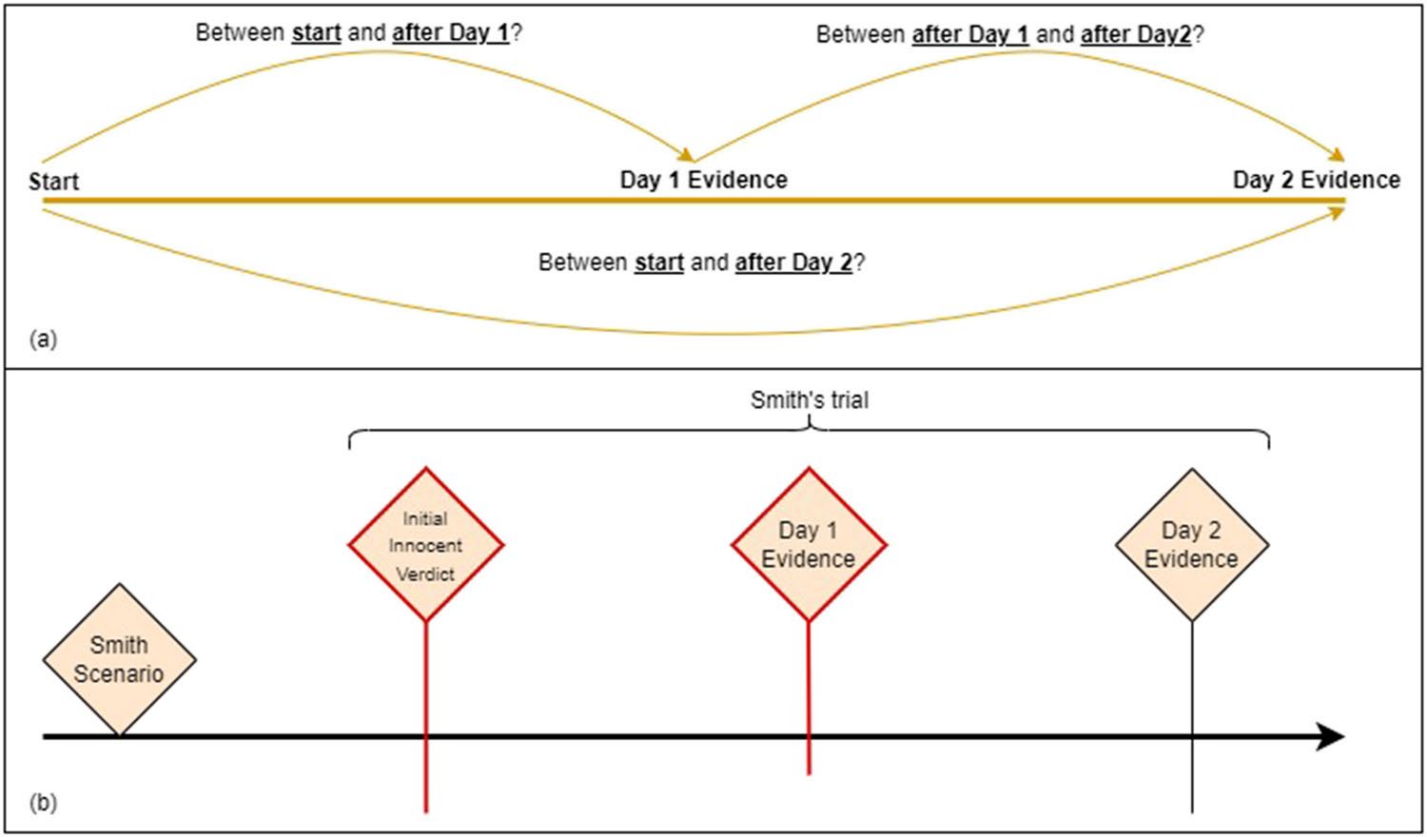
The two panels show the illustrations to explain the three time points in the murder case and the three change judgments. The top and bottom panels correspond to Experiment 1 and 2, respectively. In Experiment 2, participants first saw an illustration of the three time points during the trial and subsequently the same illustration was employed to highlight the particular change judgment requested from a participant

In both experiments, we explained to participants the idea of a change measurement by telling them, “Imagine being asked whether you are feeling colder or warmer. Sometimes you can answer such a question, without considering whether you are cold or warm in some absolute sense. ….” In Experiment 1, the specific question participants saw was, for example, “Would you say you changed your mind about Smith’s guilt between start and after Day 1?” and the Fig. 3a illustration was offered. In Experiment 2, participants were additionally told “In the main part of the experiment, we will ask you at some point for a change judgment, for the guilt versus innocence of a hypothetical suspect. What matters is not whether you consider the person guilty or innocent, but whether your verdict has changed (for example, whether you considered the person initially innocent but now you think he is guilty; or the other way round!).” In Experiment 2, the change judgment was phrased as, for example, “Did you change your verdict between your initial innocent verdict and after reading the day 1 evidence?”, and the illustration employed was as in Fig. 3b. Each participant responded to only one change question, i.e., the three possible change questions, *C*_*start*, *day*1_, *C*_*start*, *day*2_, *C*_=*day*1, *day*2_, defined the three between participants conditions in the experiment.

In both experiments, participants initially received instructions outlining the idea of a change judgment and then saw the information about Dixon and the trial for Smith, followed by the day 1 evidence. Subsequently, participants in the *C*_*start*, *day*1_ condition were shown a few multiple choice questions about the presented information, which had to be answered correctly before proceeding, then were asked for the change judgment (yes/no), then saw the day 2 evidence, and then received two further, free-text questions, asking them about the presented evidence on the two days. Participants in the *C*_*start*, *day*2_, *C*_*day*1, *day*2_ conditions first saw the day 2 evidence, before proceeding as above.

Subsequently, participants went through a similar hypothetical trial, but in which they were asked for two individual judgments of guilt, at two separate time points. The purpose of these additional judgments was to obtain reaction time (RT) data for individual judgments, for a comparison with RTs for change judgments. Specifically, participants were told to imagine that another suspect has been charged with murder. A slightly different cover story was employed (details in the OSF project page). However, rather than asking participants for a change judgment, we instead asked them for a verdict for the suspect, based on the day 1 evidence. Participants then read about a third hypothetical crime, analogous to the previous one, but with changes in the names and other minor details. Participants were then asked for a verdict on the basis of the evidence from both day 1 and day 2.

Finally, participants were debriefed. The experiments lasted about 10 min.

## Results

A valid test of a TB inequality violation presupposes an ensemble of identically prepared “systems.” In physics, this is straightforward, since precise engineering can guarantee a sample of such systems. In behavioral sciences, the only way forward is to ensure that unexcluded participants are uniform, in terms of their processing of the relevant information. In both experiments, participants provided three free-text answers, for their decision and memory of the evidence. We adopted a set of criteria for excluding participants who offered inadequate responses (Table 2; OSM 3).

**Table 2:** Exclusions due to information processing failures

	*N*_−_(*t*_1_, *t*_3_)	*N*_−_(*t*_1_, *t*_2_)	*N*_−_(*t*_2_, *t*_3_)
Experiment 1	42 out of 133	28 out of 135	38 out of 133
Experiment 2	51 out of 133	31 out of 139	52 out of 137

In each cell, “out of” indicates the recruited participants prior to exclusions

We have seen that Eq. 2 can be rewritten in terms of frequencies of change judgments, as *N*_−_(*t*_1_, *t*_3_) ≤ *N*_−_(*t*_1_, *t*_2_) + *N*_−_(*t*_2_, *t*_3_), whereby, for example, *N*_−_(*t*_1_, *t*_3_) indicates the number of participants responding that their judgment changed between start and after the second day evidence. This can be translated to a comparison of proportions, testing whether the quantity *tb* = *pr*_−_(*t*_1_, *t*_3_) − *pr*_−_(*t*_1_, *t*_2_) − *pr*_−_(*t*_2_, *t*_3_) is greater than 0, where *pr* refers to proportions. The standard error of the mean (*se*) for binary categorical data is given by $SE=\sqrt{\frac{\textrm{pq}}{\textrm{n}}}$, where *p*, *q* are the probabilities of the two outcomes. The pooled *se* for *tb* is given by ${SE}_{pooled}=\sqrt{\sum_i{\left(\frac{\partial TB}{\partial {x}_i}\right)}^2{\left({SE}_i\right)}^2}$, whereby *x*_*i*_ are the coefficients to each proportion. That is, ${SE}_{pooled}=\sqrt{SE{\left({t}_1,{t}_3\right)}^2+ SE{\left({t}_1,{t}_2\right)}^2+ SE{\left({t}_2,{t}_3\right)}^2}$. Note, this is an upper bound for *SE*_*pooled*_, which does not take into account the covariance between the three proportions. We can now construct 95% confidence intervals (95% CIs) for *tb* as *tb* ± 1.96 ∙ *SE*_*pooled*_.^2^ A violation of the TB inequality requires *tb*>0 and so our approach to significance rests on whether the 95% CI for *tb* includes 0 or not.

We first discuss Experiment 1 results. The *tb* quantity offers no evidence for a TB inequality violation (Table 3).

**Table 3:** Change decisions, in Experiments 1 and 2

	*N*_−_(*t*_1_, *t*_3_)	*N*_−_(*t*_1_, *t*_2_)	*N*_−_(*t*_2_, *t*_3_)	*tb*
Exp. 1	7 out of 91	2 out of 107	7 out of 95	-0.02, LB=-0.10, UB=0.06
Exp. 2	14 out of 82	4 out of 108	4 out of 85	0.09, LB=-0.01, UB=0.19

For the first three columns, each cell shows change decisions out of the total participants in the condition (each cell is a between participants condition). The fourth column shows the *tb* quantity, *tb* = *pr*_−_(*t*_1_, *t*_3_) − *pr*_−_(*t*_1_, *t*_2_) − *pr*_−_(*t*_2_, *t*_3_), and 95%CI bounds (LB: lower bound, UB: upper bound)

We next compared reaction times between the change judgment and the two individual judgments each participant provided. It is possible that a change judgment consists of two separate judgments and a calculation of difference, in which case a change judgment should take longer than any individual judgment (more than twice as long). A one-way within-participants ANOVA on reaction times with a single factor of judgment (change, initial, subsequent) was significant, *F*(1.871,557.537) = 5.549, *p* = .005, using the Greenhouse-Geisser correction (note, here and just below, the change judgment was introduced as a single variable, without consideration of the two time points across which the change occurred). Uncorrected post-hoc, two-tailed t-tests, showed that the mean reaction time for the change judgment (average value per participant *M* = 7.51 s) was equivalent to that of the initial individual judgment (*M* = 7.0 s, *t*(300) = 1.251, *p* = .212), but higher compared to the subsequent individual judgment (*M* = 6.2 s, *t*(300) = 2.743, *p* = .006). We cannot conclude whether a change judgment is composed of two individual judgments or not.

In Experiment 2, the *tb* quantity is consistent with a TB inequality violation, though the lower 95% CI bound is just below 0 (Table 3). We examined whether this conclusion depends on the exclusion criteria. Across two reasonable variations of the criteria, in one case the results were nearly identical to those in Table 3 and in the other case there was still a trend for a violation, but with weaker evidence. We also considered change statistics without any exclusion criteria. This analysis included participants who committed obvious errors in the free text questions about the presented evidence, for example, that there was “no evidence” for guilt in day 1. With this analysis, there was no evidence for a TB inequality violation, but we do not think this is a valid approach (OSM 3). We cannot unambiguously conclude that there is a TB inequality violation, though there is good indication that this is so.

Regarding reactions times, in this case, there is data only for the first sampling part of Experiment 2. Reaction times for both individual questions (*M* = 6.52, *M* = 4.95) were significantly shorter than reaction times for the change question (average *M* = 8.99). The overall ANOVA was significant, *F*(2, 272) = 18.2, *p* < .001, as were pairwise comparisons between mean for the change judgment and the first individual judgment (*t*(136) = 4.105, *p* < .001) and between the change judgment and the second individual judgment (*t*(136) = 5.679, *p* < .001). Given that the individual judgments were requested after the change one, it is possible that the lower reaction times for the former reflect practice effects. We ran a pilot experiment to address this possibility and, with putative practice effects eliminated, we found that the reaction time for an individual judgment was indistinguishable to that for a change one (OSM 3).

For both experiments one might ask whether alternative versions of the TB inequality are violated – there are four versions (Halliwell, 2016). Presently, we focused on the version which best matched our empirical paradigm: given the information participants received, we expected small changes between time points 1, 2 and 2, 3 and a larger change between time points 1, 3. So, observing a violation for any of the other three versions would have been dubious. Additionally, it can be shown that violating one version of the TB inequalities precludes violations of any other versions (OSM 2).

## Discussion

The observed violation of the TB inequality is evidence against macrorealism, that is, the assumption that a question can have a specific value (even if unrevealed) at all time.^3^ Without macrorealism, resolving a question may change mental representations, giving rise to order or interference effects. In both memory (Howe, 2011; Schacter et al., 2011) and generally (Hogarth & Einhorn, 1992; Schwarz, 2007; Sharot et al., 2010), there have been arguments of constructive processes, so what is the added value of an examination involving the unfamiliar framework of the TB inequality?

The problem is that there are many possible ways in which a recollective process or judgment can impact on the mental representations. A measurement that is too coarse (a “sledgehammer” measurement) is likely to change the relevant system, but a corresponding conclusion would simply tell us that greater care is needed with our measurement approach. In physics, the NIM assumption is one which tests whether a measurement is sufficiently “adroit” to prevent disturbance of a target system (Wilde & Mizel, 2012). However, in physics it has been hotly debated whether it is possible to test for violations of the TB inequality, while conforming to the NIM assumption, because quantum measurements can also disturb a system (Emary, 2017; Emary et al., 2015; Halliwell, 2016). In behavioral sciences, we can get round this problem, by requesting change judgments, which can be directly related to the quantities needed to test for violations of a TB inequality. Note, it is still possible that, even when a decision is not directly made, participants implicitly decide on guilt vs. innocence, every time new information is offered. More work is needed to address such possible loopholes.

If we can make the NIM assumption, a violation of the TB inequality can be interpreted as evidence for quantum-like structure in human memory processes. To the best of our knowledge, this is the first study offering some evidence for a TB inequality violation, based on change judgments, which offer reasonable protection regarding the NIM requirement. As discussed, whether constructive processes in recollection are quantum-like vs. not is a key issue, since the former possibility allows us to constrain the nature of such processes, in terms of the operations allowed in quantum theory (cf. White et al., 2020). Quantum-like memory models and ideas have already been explored in memory (Brainerd et al., 2013; Manning, 2021; Trueblood & Hemmer, 2017), though the use of the TB inequality offers a more general/ generic test of these ideas.

Our work shows that, despite the conceptual simplicity of TB inequality tests, there are subtle methodological challenges. The situation is analogous to that in physics, whereby, even though Bell carried out his pioneering work in 1960s, it took decades of intense work (culminating in the Nobel prize in physics, in 2022) before a convincing violation was demonstrated. Specifically, the psychological analogue of identically prepared systems required heavy-handed exclusion criteria, which might be uncommon in other areas of our field. We think that the most justified exclusion approach offers statistical evidence for a TB Inequality violation and, moreover, across reasonable variants of the exclusion approach, there is a consistent trend for a violation (though in one case, the statistical evidence is weak and if all participants are included, there is no evidence for a violation). Note, even if we assume that participants process the information diligently, they are unlikely to be exactly “identical.” Can TB inequality violations arise incidentally from participant differences? If we could prepare any mixture of systems (e.g., a group of similar, but non-identical participants), and measure in a non-disturbing way, the change statistics should still satisfy the TB inequalities (this is because TB inequalities are a linear functional on a system). But, sampling variation could result in caveats in this picture, if, for example, participants in one condition have a stronger bias to report changes. Overall, an empirical challenge for future work is how to balance the competing prerogatives of large enough expectations for change judgments, homogeneous participants, and ceiling effects in overall change.

Future work should further explore why evidence for quantum-like structure in memory is significant. We can consider a recollection as a correlation between queries at present and memories for past events. We can then ask about the maximum correlation for mappings, between queries and events, if we assume classical resources (classical probability theory) or quantum-like resources. As it turns out, utilizing quantum-like resources leads to higher correlations, than with classical resources (Budroni et al., 2019). That is, a memory system with quantum-like representations may allow more efficient recollective processes. More work is needed to substantiate this proposal, but the essential idea can be explained simply. Classically, a conjunction can never be higher than a marginal, but behaviorally people often conclude that *p*(*A* & *B*) > *p*(*B*), a famous finding in both decision-making (Tversky & Kahneman, 1983) and memory (Brainerd & Reyna, 2008). Classically, we can avoid an incorrect judgment by the inclusion of a conditionalizing parameter (*p*(*A* & *B*| *x*) > *p*(*B*)), thereby employing more resources. In quantum theory, we can immediately allow *p*(*A* & *B*) > *p*(*B*), without additional resources. If a cognitive agent lives in a world where she encounters plenty of instances of *p*(*A* & *B*) > *p*(*B*), then it makes sense to employ quantum-like representations, instead of classical ones (this might be the case if, e.g., measurements change the system; Pothos et al., 2017). A similar analogy can be built in relation to temporal/ memory situations, though precisely how is the topic of future work.

In conclusion, an examination of the TB inequality revealed a novel way to advance our understanding of constructive processes in memory. The observed violation of the TB inequality increases confidence that quantum-like representations are a plausible way to understand some aspects of human behavior (Manning, 2021).

### Supplementary information

ESM 1 (DOCX 80 kb)

## Footnotes

1 Noninvasive measurability means that the marginal distribution at each point should not change. Specifically, we do not want the marginal distribution at *t*_2_ to change, depending on a measurement at *t*_1_ (i.e., we want to avoid “signaling”).

2 Note, we also employed a bootstrapping approach for the calculation of the confidence intervals, rather than rely on the 95% CI formula in text (which assumes a normal sampling distribution), and the values we obtained were nearly identical to the ones from the formula.

3 This distinction encompasses classical linear mixtures too, for example, 35% guilty, 65% innocent. As discussed, these can be meaningfully distinguished from quantum superpositions.
