# Supplementary material for: Temporal Bell inequalities in cognition
Source: Psychon Bull Rev. 2023 Apr 17;30(5):1946–53. doi: 10.3758/s13423-023-02275-5 (PMC10716061; doi:10.3758/s13423-023-02275-5)
Supplement: Supplementary file 1 — (DOCX 80 kb) [file 13423_2023_2275_MOESM1_ESM.docx]

Supplementary Material 1

As mentioned in main text, the way giving up macrorealism offers a puzzling view of behavior can be illustrated by using an alternative form of the TB inequality, expressed in terms of the number of times an observable changes across specific time points (Atmanspacher & Filk, 2010). For example, $N_{-}\left( t_{1},t_{2} \right)$ tells us the number of times the binary question (observable) has changed across the first and second time points, across all possible values that the question can have at these time points. If, for example, at t1 the question is + and at t2 -, then we add one to $N_{-}\left( t_{1},t_{2} \right)$. Table 1 shows all the possibilities for what $N_{-}\left( t_{1},t_{2} \right)$, $N_{-}\left( t_{1},t_{3} \right)$, $N_{-}\left( t_{2},t_{3} \right)$ can be, depending on the values of the questions at the three time points. It is a straightforward fact of set theory that $N_{-}\left( t_{1},t_{3} \right)\leq N_{-}\left( t_{1},t_{2} \right)+N_{-}\left( t_{2},t_{3} \right)$ and this inequality is equivalent to the TB one (we show this directly below; see also Atmanspacher & Filk, 2010; Yearsley & Pothos, 2014).

The violation of the TB inequality, which occurs when $N_{-}\left( t_{1},t_{3} \right)>N_{-}\left( t_{1},t_{2} \right)+N_{-}\left( t_{2},t_{3} \right)$, requires that we may have few changes across *t*_1_, *t*_2_ time points, few changes across *t*_2_, *t*_3_ time points, but numerous changes across the *t*_1_, *t*_3_ time points. Clearly, if the question has fixed (independent of measurement) answers across the three time points, this is impossible. So, the TB inequality is essentially about the way a cognitive variable changes across time. If changes are well-behaved, the number of changes across *t*_1_, *t*_2_ and *t*_2_, *t*_3_ should be greater than across *t*_1_, *t*_3_. If the TB inequality is violated, then “the violation of a TB inequality involving a particular observable, at different time points, implies that it is impossible to have a joint probability distribution for the (assumed possessed) value of the observable across all these time points ... to concurrently fix the observable values across all time points” (Yearsley & Pothos, 2014, p.7).

| s(t_1_) | s(t_2_) | s(t_3_) | *N*_(t_1_,t_3_) | *N*_(t_1_,t_2_) | *N*_(t_2_,t_3_) |
| --- | --- | --- | --- | --- | --- |
| +1 | +1 | +1 |  |  |  |
| +1 | +1 | -1 | x |  | x |
| +1 | -1 | +1 |  | x | x |
| +1 | -1 | -1 | x | x |  |
| -1 | +1 | +1 | x | x |  |
| -1 | +1 | -1 |  | x | x |
| -1 | -1 | +1 | x |  | x |
| -1 | -1 | -1 |  |  |  |

Table 1. Values of the binary observable/ question at the three time points, *t*_1_, *t*_2_, *t*_3_ and the corresponding change statistics.

The above readily suggests that change measurements are a valid way to assess the TB inequality, but it would be reassuring to show this directly. Recall that the TB inequality is $C_{ab}+C_{bc}\leq C_{ac}+1$, such that e.g. $C_{ab}=\Pr\left( ++ | a,b \right)\cdot1\cdot1+\Pr\left( -- | a,b \right)\cdot\left( -1 \right)\cdot\left( -1 \right)+\Pr\left( +- | a,b \right)\cdot1\cdot\left( -1 \right)+\Pr\left( -+ | a,b \right)\cdot\left( -1 \right)\cdot1$. That is, the quantities $C_{ab}$ can be understood as correlations. From just above, from Atmanspacher and Filk (2010), we know that we can express the TB inequality in terms of number of changes across different time points, $N_{-}\left( t_{1},t_{3} \right)\leq N_{-}\left( t_{1},t_{2} \right)+N_{-}\left( t_{2},t_{3} \right)$. We next explain in more detail how the relation between the standard correlators in the TB inequality and the change measurements employed in the empirical paradigm come about.

Let us introduce some simple notation, namely that the suspect on each day is either innocent, $v_{d}=+1$, or guilty, $v_{d}=-1$, where ‘v’ stands for ‘value’ and ‘d’ stands for ‘day’. The correlations between two days x, y are then computed as $C_{xy}=\sum_{v_{x},v_{y}} v_{x}v_{y}P\left( v_{x},v_{y} \right)=\sum_{v_{x}=v_{y}} v_{x}v_{y}P\left( v_{x},v_{y} \right)+\sum_{v_{x}\neq v_{y}} v_{x}v_{y}P\left( v_{x},v_{y} \right)$. When $v_{x}=v_{y}$, $v_{x}v_{y}=+1$ and when $v_{x}\neq v_{y}$, $v_{x}v_{y}=-1$. So, we have $C_{xy}=\sum_{v_{x}=v_{y}} P\left( v_{x},v_{y} \right)-\sum_{v_{x}\neq v_{y}} P\left( v_{x},v_{y} \right)$. So far, we have just expressed the expectation value concerning the judgments across the two days, x, y, in a way exactly analogous to what we have done above.

We are next interested in the change from day x to day y, which we can quantify as $\Delta=+1$ for change and $\Delta=-1$ for no change. Then, the expectation for change can be written as $\left\langle\Delta\right\rangle=-1\sum_{v_{x}=v_{y}} P\left( v_{x},v_{y} \right)+1\sum_{v_{x}\neq v_{y}} P\left( v_{x},v_{y} \right)$.

It follows that $C_{xy}=-\left\langle\Delta\right\rangle$, when a change is encoded as $\Delta=+1$ for change and $\Delta=-1$ for no change. Note that the key issue is just whether there is a change or not, not the direction of the change. If change is encoded instead so that $\Delta'=+1$ for change and $\Delta^{'}=0$ for no change, we simply have $\Delta=2\Delta^{'}-1$ and so $C_{xy}=-2\left\langle\Delta^{'} \right\rangle+1$. Adopting this latter approach, $C_{ab}+C_{bc}\leq C_{ac}+1\Longleftrightarrow-2\left\langle{\Delta^{'}}_{ab} \right\rangle+1-2\left\langle{\Delta^{'}}_{bc} \right\rangle+1\leq-2\left\langle{\Delta^{'}}_{ac} \right\rangle+1+1\Longleftrightarrow\left\langle{\Delta^{'}}_{ab} \right\rangle+\left\langle{\Delta^{'}}_{bc} \right\rangle\geq\left\langle{\Delta^{'}}_{ac} \right\rangle$. The latter inequality is closely related to the one $N_{-}\left( t_{1},t_{3} \right)\leq N_{-}\left( t_{1},t_{2} \right)+N_{-}\left( t_{2},t_{3} \right)$ from Atmanspacher and Filk (2010).

Note a slightly curious aspect of this formulation, namely that this picture does not distinguish between whether the change is one of assuming initially the suspect is guilty and change to assume he is less guilty or assuming initially the suspect is innocent and change to assume he is less innocent. That is, consider asking participants:

$\Delta^{''}=+1$ – the suspect is judged innocent compared to a guilty verdict before.

$\Delta^{''}=-1$ – the suspect is judged guilty compared to an innocent verdict before. But the distinction between change towards guilt vs. change towards innocence does not matter, because the $C_{xy}$ values are sensitive only to whether there is change vs. not. Therefore, $\Delta^{''}$ can just be recast onto a variable analogous to $\Delta^{'}$ and the above formula for conversion to $C_{xy}$ be employed.

Supplementary Material 2

We provide some indicative calculations showing how the Figure 2 illustration can translate to a more concrete model. These calculations assume some familiarity with the basics of quantum theory. In main text, Figure 2 was put together by analogy to the empirical demonstration and we offer the calculation with reference to Figure 2.

A trial involves an initial state such that participants assume initial innocence regarding the guilt/ innocence of the suspect, some evidence on day 1 and some evidence on day 2. Therefore, the three time points required for a TB setup are {time 1 (a), time 2 (b), time 3 (c)}= {initial state, day 1 evidence, day 2 evidence}. Recall that the temporal Bell inequality is $C_{ab}+C_{bc}\leq C_{ac}+1$. The assumption of initial innocence means that the initial state is $\psi=\left. |innocent \right\rangle$.

Assume one-dimensional observables for simplicity of illustration (so we can easily generate diagrams as in Figure 2). Then, $C_{ab}=\Pr\left( ++ | a,b \right)\cdot1\cdot1+\Pr\left( -- | a,b \right)\cdot\left( -1 \right)\cdot\left( -1 \right)+\Pr\left( +- | a,b \right)\cdot1\cdot\left( -1 \right)+\Pr\left( -+ | a,b \right)\cdot\left( -1 \right)\cdot1$.

$\Pr\left( ++ | ab \right)=\Pr\left( ++ | initial,Day1 \right)$ is the joint probability of having a + (say innocent) initially and a + on Day1 (Figure 2). To compute $\Pr\left( ++ | ab \right)$, we have to measure whether the participant considers the suspect to be innocent or not against the initial state, rotate the resulting state by angle $\theta_{ab}$ (this is the change from time *a* to time *b*), and then also measure whether we have innocent or not. Note, in this picture we assume that the question is the same across the three time points and what changes is the state. Note also that this is a counterclockwise rotation and, since the clockwise rotation operator is $\left( \begin{matrix} \cos ab & -\sin ab \\ \sin ab & \cos ab \end{matrix} \right)$, the counterclockwise rotation operator would be $\left( \begin{matrix} \cos ab & \sin ab \\ -\sin ab & \cos ab \end{matrix} \right)$. We can, instead, assume that the state is the same but the observable changes – these two pictures are equivalent and preference for one vs. the other is a matter of convenience.

We make the simplifying assumption that $\theta_{ab}=\theta_{bc}=\theta$ and also note that $\left. |day1 \right\rangle=U_{\theta}\left. |innocent \right\rangle=\left( \begin{matrix} \cos\theta& -\sin\theta\\ \sin\theta& \cos\theta\end{matrix} \right)\left( \begin{matrix} 1 \\ 0 \end{matrix} \right)=\left( \begin{matrix} \cos\theta\\ \sin\theta\end{matrix} \right)$ and $\left. |day2 \right\rangle=U_{2\theta}\left. |innocent \right\rangle=\left( \begin{matrix} \cos2\theta\\ \sin2\theta\end{matrix} \right)$. Also note that $P_{innocent}=\left( \begin{matrix} 1 & 0 \\ 0 & 0 \end{matrix} \right)$ and $P_{\sim innocent}=I-\left( \begin{matrix} 1 & 0 \\ 0 & 0 \end{matrix} \right)=\left( \begin{matrix} 0 & 0 \\ 0 & 1 \end{matrix} \right)$. Then,

$$Prob\left( ++,|start, day1 \right)={|P_{innocent}U_{\theta_{ab}}P_{innocent}\left. |innocent \right\rangle|}^{2}={|\left( \begin{matrix} 1 & 0 \\ 0 & 0 \end{matrix} \right)\left( \begin{matrix} \cos\theta& -\sin\theta\\ \sin\theta& \cos\theta\end{matrix} \right)\left( \begin{matrix} 1 & 0 \\ 0 & 0 \end{matrix} \right)\left( \begin{matrix} 1 \\ 0 \end{matrix} \right)|}^{2}={|\left( \begin{matrix} 1 & 0 \\ 0 & 0 \end{matrix} \right)\left( \begin{matrix} \cos\theta\\ \sin\theta\end{matrix} \right)|}^{2}=\left( \cos\theta\right)^{2}$$

which is the expected result. This can be more easily computed as

$$Prob\left( ++|start, day1 \right)={|\left\langle day 1 \right.\left. |innocent \right\rangle|}^{2}={|\left\langle day 1 \right.\left. |innocent \right\rangle|}^{2}=\left( \cos\theta\right)^{2}$$

It is straightforward to compute the remaining probabilities as

$$\Pr\left( -- | start, day1 \right)={|\left( \begin{matrix} 0 & 0 \\ 0 & 1 \end{matrix} \right)\left( \begin{matrix} \cos\theta& -\sin\theta\\ \sin\theta& \cos\theta\end{matrix} \right)\left( \begin{matrix} 0 & 0 \\ 0 & 1 \end{matrix} \right)\left( \begin{matrix} 1 \\ 0 \end{matrix} \right)|}^{2}=0$$

$$\Pr\left( +- | start, day1 \right)={|\left( \begin{matrix} 0 & 0 \\ 0 & 1 \end{matrix} \right)\left( \begin{matrix} \cos\theta& -\sin\theta\\ \sin\theta& \cos\theta\end{matrix} \right)\left( \begin{matrix} 1 & 0 \\ 0 & 0 \end{matrix} \right)\left( \begin{matrix} 1 \\ 0 \end{matrix} \right)|}^{2}={|\left( \begin{matrix} 0 & 0 \\ 0 & 1 \end{matrix} \right)\left( \begin{matrix} \cos\theta\\ \sin\theta\end{matrix} \right)|}^{2}=\left( \sin\theta\right)^{2}$$

$$\Pr\left( -+ | start, day1 \right)={|\left( \begin{matrix} 1 & 0 \\ 0 & 0 \end{matrix} \right)\left( \begin{matrix} \cos\theta& -\sin\theta\\ \sin\theta& \cos\theta\end{matrix} \right)\left( \begin{matrix} 0 & 0 \\ 0 & 1 \end{matrix} \right)\left( \begin{matrix} 1 \\ 0 \end{matrix} \right)|}^{2}=0$$

Therefore, $C_{ab}={cos}^{2}\left( \theta\right)-{sin}^{2}\left( \theta\right)=cos\left( 2\theta\right)$ and, analogously, it can be shown that $C_{bc}=C_{ab}$. To compute $C_{ac}$, as above, we have

$$Prob\left( ++,|start, day2 \right)={|P_{innocent}U_{\theta_{ac}}P_{innocent}\left. |innocent \right\rangle|}^{2}=\left( \cos2\theta\right)^{2}$$

It is straightforward to see that the rest of the calculations are unchanged, with the exception that the angles are, instead of $\theta$, $2\theta$, so that $C_{ac}=cos\left( 4\theta\right)$.

Consider the Temporal Bell inequality again, which is $C_{ab}+C_{bc}\leq C_{ac}+1$. There is a violation when $C_{ab}+C_{bc}>C_{ac}+1$ or $C_{ab}+C_{bc}-C_{ac}-1>0$. This can be rewritten with the expressions above, as: 2$\cdot cos(2\theta)-cos(4\theta)-1>0$. The meaningful range for $\theta$ here is $\left[ 0,\pi/4 \right]$, since $\theta_{ab}+\theta_{bc}$ has to be less than $\pi/2$. In this range, we always get a violation of the Temporal Bell inequality (Figure 1S). Overall, we can see that, as long as quantum-like representations are employed and the structure of the problem approximately conforms to that of Figure 2, quantum theory predicts a violation of the TB inequality.


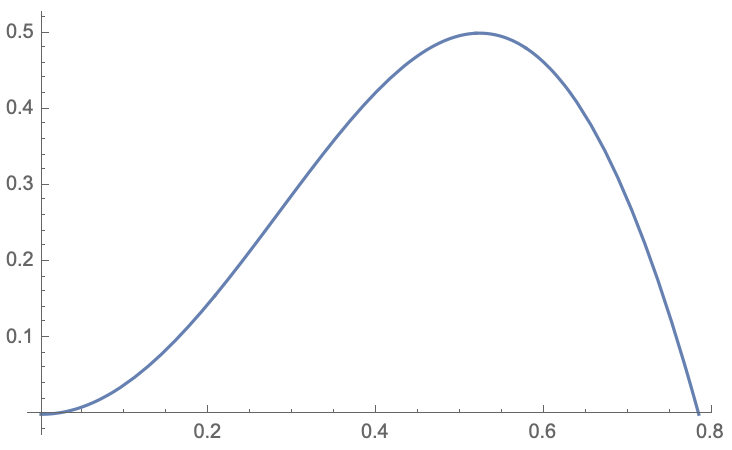


Figure 1S. The extent of violation of the TB inequality. We are plotting the quantity $2C_{ab}-C_{ac}-1$ on the vertical axis, while on the horizontal axis we have the size of the angle $\theta$. Positive values indicate a violation of the TB inequality.

A final point to consider is whether it is possible to violate multiple variants of the TB inequality at the same time, but this is actually not possible. We offer a simple proof of this point as follows:

Consider the four TB inequality expressions

$\boldsymbol{C}_{1}:=C_{12}+C_{13}-C_{23}$ (S1.1)

$\boldsymbol{C}_{2}:=C_{12}-C_{13}+C_{23}$ (S1.2)

$\boldsymbol{C}_{3}:=-C_{12}+C_{13}+C_{23}$ (S1.3)

$\boldsymbol{C}_{4}:=-C_{12}-C_{13}-C_{23}$ (S1.4)

where $C_{ij}$ are the correlates of the measurement results in the respective times *i* and *j*. Then the four TB inequalities take the form

$\boldsymbol{C}_{k}\leq1$ for *k*=1,2,3,4 (S1.5)

which, as noted, determine whether joint probability distributions for the outcomes in three time points exist or not. We note that, in principle, each of the TB inequality expressions in Eq. (5) can take a value of up to 3, since $|C_{23}|\leq1$. However, the particular algebraic structure of these equations implies that:

**Fact**. *For any observed statistic at most one out of the four TB inequalities in Eq. (5) can be violated at the same time, i.e., it is not possible that any two expressions in Eqs. (1) – (4) can be simultaneously larger than 1.*

**Observation 1**. We have that $\boldsymbol{C}_{k}+\boldsymbol{C}_{l}\leq2$ whenever $k\neq l$.

*Proof*. Observe that each expression $\boldsymbol{C}_{1},\ldots\boldsymbol{C}_{4}$ is a particular combination of three numbers, *a*, *b*, and *c* in the form

$\boldsymbol{C}_{1}=a+b-c$, $\boldsymbol{C}_{2}=a-b+c$, $\boldsymbol{C}_{3}=-a+b+c$, $\boldsymbol{C}_{4}=-a-b-c$ (S1.6)

for some $-1\leq a,b,c\leq1$. Now add up these equations in pairs to obtain

$\boldsymbol{C}_{1}+\boldsymbol{C}_{2}=\left( a+b-c \right)+\left( a-b+c \right)=2a\leq2$ (S1.7)

$\boldsymbol{C}_{1}+\boldsymbol{C}_{3}=\left( a+b-c \right)+\left( -a+b+c \right)=2b\leq2$ (S1.8)

$\boldsymbol{C}_{1}+\boldsymbol{C}_{4}=\left( a+b-c \right)+\left( -a-b-c \right)=-2c\leq2$ (S1.9)

$\boldsymbol{C}_{2}+\boldsymbol{C}_{3}=\left( a-b+c \right)+\left( -a+b+c \right)=2c\leq2$ (S1.10)

$\boldsymbol{C}_{2}+\boldsymbol{C}_{4}=\left( a-b+c \right)+\left( -a-b-c \right)=-2b\leq2$ (S1.11)

$\boldsymbol{C}_{3}+\boldsymbol{C}_{4}=\left( -a+b+c \right)+\left( -a-b-c \right)=-2a\leq2$ (S1.12)

Since in each case four out of six terms cancel out and we have $-1\leq a,b,c\leq1$.

Supplementary Material 3

In this section, we consider some additional empirical details.

First, we outline the exclusion criteria employed for the two experiments. Note, as briefly mentioned in text, a fundamental assumption for TB inequality tests in physics is that the tests are carried out on an ensemble of identically prepared systems. In behavioral sciences, it is impossible to exactly fulfil this assumption. However, with clearly set exclusion criteria, we can at least increase our confidence that the retained participants are ones who processed the information in the intended way.

The exclusion criteria concerned the three free-test questions participants were asked, that is, to justify their decision and to state their memory of the day 1 and day 2 evidence. The criteria were: errors in the way the information was recalled, including whether a pill bottle was mentioned (instead of an alcohol bottle), as well as more obvious errors or failure to offer any information for one of the three questions; duplicating information across any of the three answers; outlier completion times (only a handful – less than 5 – participants were eliminated in this way in both experiments); failure to mention either Smith or Dixon at least once, across the three answers; a mention of just ‘fingerprints’ in the answers. These exclusion criteria led to Tables 2, 3 in main text.

A reasonable question is whether altering these criteria forces us to revise our conclusion (we focused on Experiment 2, since this is the case of interest). One alternative approach is this: even though some participants failed to mention either Dixon or Smith in any of their three answers, they still offered (comparatively speaking) eloquent text. So, a subjective judgment was made to retain such responses, leading to an additional 10 participants being retained, see Table 3 variant (across all conditions; recall, total sample was 409 participants in Experiment 2). In such a case, the results hardly change.

Table 3 variant. Change decisions, in Experiment 2, retaining ‘expressive’ participants.

$N_{-}\left( t_{1},t_{3} \right)$ $N_{-}\left( t_{1},t_{2} \right)$ $N_{-}\left( t_{2},t_{3} \right)$ *tb*

Exp. 2 14 out of 87 4 out of 108 4 out of 90 0.08, LB=-0.02, UB=0.17

Another alternative approach is to completely eliminate the exclusion criterion based on mentioning Smith or Dixon at least one – note, we do think that this criterion is justified, because it is an important aspect of the information provided. In this case, 72 participants were eliminated in total, out of 409.

Table 3 second variant. Change decisions, in Experiment 2, retaining participants who failed to mention either Smith or Dixon in any of the three free-text answers.

$N_{-}\left( t_{1},t_{3} \right)$ $N_{-}\left( t_{1},t_{2} \right)$ $N_{-}\left( t_{2},t_{3} \right)$ *tb*

Exp. 2 16 out of 102 4 out of 122 8 out of 113 0.05, LB=-0.04, UB=0.14

With this second variant concerning exclusion criteria, the evidence concerning a violation of the TB inequality is ambiguous (Table 3 second variant). A final variant to the analysis concerns change statistics without any exclusion criteria at all, that is, including all 409 participants in Experiment 2. We think that this is a not a valid approach, as it included participants who committed obvious errors in the free text questions corresponding to the evidence presented across the two days of the trial. However, we offer the results, for completeness. It can be seen that, when including all participants, there is no evidence for a violation of TB inequalities.

Table 3 third variant. Change decisions, in Experiment 2, retaining all participants, regardless of evidence of misunderstanding/ lack of attention.

$N_{-}\left( t_{1},t_{3} \right)$ $N_{-}\left( t_{1},t_{2} \right)$ $N_{-}\left( t_{2},t_{3} \right)$ *tb*

Exp. 2 24 out of 133 7 out of 139 17 out of 137 0.01, LB=-0.09, UB=0.10

The issues here reveal inherent limitations in a behavioral translation of the physics paradigm for TB inequality violations. Psychologically, it makes sense to ensure that the change events are relatively rare, to avoid ceiling effects across the extreme time points. In physics, this would not have been not a problem, because TB inequality tests can involve ensembles of relevant systems (e.g., microscopic particles), whose size is tens or hundreds of thousands. In behavioral sciences, it is impractical to recruit more than a few hundred participants. This means that results are more vulnerable to the particulars of exclusion details, which is undesirable. A major challenge for future work is how to balance the expectation for change judgments with other considerations, so as to fine-tune the strength of empirical tests for TB inequality violations.

Notwithstanding these points, in all reasonable cases there is a clear trend for a violation of the TB inequality in Experiment 2. Additionally, employing the exclusion approach which we think is most justified and objective, the evidence favors a conclusion of TB inequality violation (with a lower 95%CI bound which is very slightly below 0).

Second, we consider evidence that a change judgment cognitively corresponds to a single judgment, rather than two separate judgments and the calculation of their difference. In Experiments 1 and 2, we attempted to establish this by having participants produce some individual judgments, after they went through the main scenario and made their change judgment. Recall, a change judgment corresponds to asking participants whether they thought their verdict changed across different time points. By contrast, we call an individual judgment a judgment concerning the evidence after a particular day, e.g., whether the suspect would be judged guilty or innocent, following the evidence after Day 2.

A proxy measure for whether a change judgment is equivalent to a single judgment is reaction times, the idea being that if the reaction time for the former is equivalent to that for the latter, then the change judgment cannot be two separate individual judgments. In Experiments 1 and 2 we could not justify this assumption, with average reaction time for individual judgments in general lower than for change judgments. However, since in these cases the individual judgments always followed the change judgment, it is possible that faster reaction times simply reflected practice effects. So, the purpose of the pilot experiment presented in this section is to evaluate the possible equivalence between change judgments and individual ones (using reaction times), with a procedure which just involves single judgments and so avoids the potential confound of practice effects.

**Participants**

We recruited 100 participants (55 males, 44 females, 1 did not say) from mTurk, restricting recruitment to ‘Master workers’. Participants were paid $1 for their time. Participants were between 28 and 78 years old (mean age = 45.72 years, standard deviation = 10.2 years). Participants reported their English fluency on a scale from 1 (extremely uncomfortable) to 5 (extremely comfortable), with almost every participant reporting extreme comfort in communicating in English (99/100).

**Materials and procedure**

We employed the same scenario and pieces of evidence which we used in Experiment 2 for the change judgment. In this case, however, this scenario and pieces of evidence were used for individual judgments only. That is, participants in this experiment were only asked to make a single judgment, either corresponding to the suspect’s guilt after the Day 1 evidence or corresponding to his guilt after the evidence for both Day 1 and Day 2. The RT for each judgment was the main recorded variable.

**Results**

The RTs for the individual judgments from this experiment were compared to the RT for the change judgment in Experiment 2. We first conducted a one-way between participants ANOVA on the change and individual judgment RTs, which was not significant, *F*(2, 374) = .067, *p* = .935. We then conducted independent samples t-tests to compare the RT for the change judgment with the RT for the Day 1 judgment and the Day 2 judgment. Both comparisons were not significant, *t*(323) = .-.135, *p* = .893; *t*(259.3) = -.656, *p* = .512 (note, for the latter test there was an adjustment for the degrees of freedom, due to a significant Levene’s test for the homogeneity of variance assumption). Next, we conducted equivalent Bayesian independent samples t-tests to examine evidence for the null hypothesis. We identified moderate evidence for the null for both comparisons, change judgment vs. Day one judgment (BF_01_ = 8.264) and change judgment vs. Day 2 judgment (BF_01_ = 7.89).

**Discussion**

When examining the RTs between a change judgment and either of the two possible individual judgments, there appears to be equivalence between the former and the latter. This result supports the assumption that a change judgment is *not* two individual judgments and a computation of a difference, which is the assumption needed to allow a conclusion of quantum-like structure from the violation of the TB inequality in Experiment 2.

A potential problem with this pilot demonstration is that the age profile of the samples across Experiment 1, Experiment 2, and the pilot is different: for the Pilot (n=100) mean age = 45.72; SD = 10.2; for Experiment 1 (n=293) mean age = 34.54; SD = 11.88; and for Experiment 2 (n=275) mean age = 44.33; SD = 10.63. We analyzed the differences in age with a one-way between participants ANOVA, which was significant, F(2,667) = 68.934, p < .001. A Tukey post-hoc test revealed that the age of the pilot experiment participants was significantly higher than the age of Experiment 1 participants (p < .001). However, the age of the pilot participants was not significantly different when compared with the age of Experiment 2 participants (p = .534). There was also a significant difference between the ages of the Experiment 2 and Experiment 1 participants (p < .001).

We think that the above results concerning age differences do not undermine our conclusions, because it is the difference between the age of participants in the pilot and in Experiment 2 that matters, since it is in Experiment 2 where we observed a TB inequality violation (in this case, ages are matched).
